# Are rhetorical commitments to adolescents reflected in planning documents? An exploratory content analysis of adolescent sexual and reproductive health in Global Financing Facility country plans

**DOI:** 10.1186/s12978-021-01121-y

**Published:** 2021-06-17

**Authors:** Asha S. George, Tanya Jacobs, Mary V. Kinney, Annie Haakenstad, Neha S. Singh, Kumanan Rasanathan, Mickey Chopra

**Affiliations:** 1 School of Public Health, University of the Western Cape, Bellville, Private Bag x17, Cape Town, 7535 South Africa; 2 Department of Global Health and Population, Harvard School of Public Health, 677 Huntington Avenue, Boston, MA 02115 USA; 3 Department of Global Health and Development, London School of Tropical Hygiene and Medicine, London, WC1E 7HT UK; 4 Health Systems Global Member, Geneva, Switzerland; 5 World Bank, 1818 H Street, NW, Washington, DC 20433 USA

**Keywords:** Adolescent health, Health financing, World Bank, Global financing facility, Development assistance, Content analysis, Multi-sectoral action, Gender, Social determinants

## Abstract

**Background:**

The Global Financing Facility (GFF) offers an opportunity to close the financing gap that holds back gains in women, children’s and adolescent health. However, very little work exists examining GFF practice, particularly for adolescent health. As momentum builds for the GFF, we examine initial GFF planning documents to inform future national and multi-lateral efforts to advance adolescent sexual and reproductive health.

**Methods:**

We undertook a content analysis of the first 11 GFF Investment Cases and Project Appraisal Documents available on the GFF website. The countries involved include Bangladesh, Cameroon, Democratic Republic of Congo, Ethiopia, Guatemala, Kenya, Liberia, Mozambique, Nigeria, Tanzania and Uganda.

**Results:**

While several country documents signal understanding and investment in adolescents as a strategic area, this is not consistent across all countries, nor between Investment Cases and Project Appraisal Documents. In both types of documents commitments weaken as one moves from programming content to indicators to investment. Important contributions include how teenage pregnancy is a universal concern, how adolescent and youth friendly health services and school-based programs are supported in several country documents, how gender is noted as a key social determinant critical for mainstreaming across the health system, alongside the importance of multi-sectoral collaboration, and the acknowledgement of adolescent rights. Weaknesses include the lack of comprehensive analysis of adolescent health needs, inconsistent investments in adolescent friendly health services and school based programs, missed opportunities in not supporting multi-component and multi-level initiatives to change gender norms involving adolescent boys in addition to adolescent girls, and neglect of governance approaches to broker effective multi-sectoral collaboration, community engagement and adolescent involvement.

**Conclusion:**

There are important examples of how the GFF supports adolescents and their sexual and reproductive health. However, more can be done. While building on service delivery approaches more consistently, it must also fund initiatives that address the main social and systems drivers of adolescent health. This requires capacity building for the technical aspects of adolescent health, but also engaging politically to ensure that the right actors are convened to prioritize adolescent health in country plans and to ensure accountability in the GFF process itself.

**Supplementary Information:**

The online version contains supplementary material available at 10.1186/s12978-021-01121-y.

## Plain English summary

The Global Financing Facility (GFF) is an initiative hosted by the World Bank aiming to increase health financing for women, children’s and adolescent health so that the poorest countries with the worst health status can reach the Sustainable Development Goals. To date very little work exists that examines the track record of the GFF in adolescent health. While adolescents are recognised as a key priority by the GFF leadership, we examine how they have been addressed in GFF country planning documents, to support improvements by national and multi-lateral actors as more countries access funding through this initiative. We reviewed the first 11 GFF Investment Cases (government planning documents) and Project Appraisal Documents (World Bank budgeted plans) available on the GFF website. We found that while several country documents signal understanding and investment in adolescents as a strategic group, this is not consistent across all countries, nor between Investment Cases and Project Appraisal Documents. In both types of documents attention to adolescents weakens as one moves from programming content to monitoring indicators to financial resources allocated. The article details both positive examples, as well as areas for improvement. Overall, while important examples of how the GFF supports adolescents exist, more must be done. Adolescents must be addressed more consistently as a core priority for the health sector. In addition, the right actors must be convened to prioritize the determinants of adolescent sexual and reproductive health in country plans and to ensure accountability in the GFF process itself.

## Background

Launched in 2015, the Global Financing Facility (GFF) aims to address the USD $33 billion annual funding gap that holds back countries with the greatest reproductive, maternal, newborn, child, adolescent health and nutrition (RMNCAH-N) needs from meeting the 2030 Sustainable Development Goals [1]. To date, 36 countries are being supported by the GFF, with funding replenished in 2018 to include all 50 countries with the greatest RMNCAH-N needs.

Hosted by the World Bank, the GFF is designed to strengthen country ownership by developing a multi-stakeholder platform and national planning process prioritising RMNCAH-N interventions and health systems investments. It aims to unlock sustainable financing by encouraging national resource mobilisation, donor coordination, and private sector contributions. Critically, funding from the GFF Trust fund can be matched by a country’s own credits in two of the World Bank’s lending mechanisms—International Development Association (IDA) and the International Bank for Reconstruction and Development (IBRD). The approach builds on the comparative advantage the World Bank has in mobilising domestic financing, given that it works directly not just with Ministries of Health, but also with Ministries of Finance [2].

Adolescence is identified by the GFF as one of the most neglected periods of life needing attention, with investment plans addressing adolescent health lauded in Bangladesh and Liberia [1]. Evidence of the nature and extent of adolescent health needs [3], as well as the soundness of investing in the health of adolescents [4], is globally recognised. Adolescents and their sexual and reproductive health are a core part of *The Global Strategy for Women’s, Children’s and Adolescents’ Health*, responding to the United Nations Every Woman and Every Child campaign [5], and prioritised by the special report by the Independent Accountability Panel monitoring global health commitments [6].

Given the recognition of adolescents as a key group central to realising RMNCAH-N goals and the momentum building behind the GFF process, we examine initial efforts of the GFF in addressing adolescent sexual and reproductive health as one part of a comprehensive agenda for realising adolescent health. Is the global policy rhetoric acknowledging the importance of adolescents and their sexual and reproductive health, matched by consistent inclusion of adolescents, their health needs and rights in country-level GFF policy documents? As a part of the Drivers Technical Working Group from Countdown to 2030 for Women’s, Children’s and Adolescents’ Health, this study undertakes an exploratory content analysis of World Bank and GFF country documents for the first eleven countries involved in the GFF to answer that question. The paper aims to further inform country and multi-lateral actors engaged with the GFF based at headquarter, regional and national levels.

## Methods

We accessed all available country planning documents from the Global Financing Facility website in 2018. This included those for Bangladesh, Cameroon, Democratic Republic of Congo (DRC), Ethiopia, Guatemala, Kenya, Liberia, Mozambique, Nigeria, Tanzania and Uganda. The documents included the Investment Cases, which are country-led plans for RMNCAH-N, and the World Bank Project Appraisal Documents (PAD), which secure financing from the World Bank for national governments supporting implementation of plans defined in the Investment Cases. Both documents are important as the Investment Case signals national priorities finalised with inputs from donors and civil society, and the latter signals funded elements by the World Bank as a key partner supporting national governments. Bangladesh was the only country which did not have an Investment Case online; and it was the only country which had two Project Appraisal Documents (one for health and one for secondary education).

While the GFF is meant to unlock additional investment, national commitments or donor agreements other than those made by the World Bank are not made available on their website.

Content analysis is a method designed to identify and interpret meaning in recorded forms of communication by isolating data that represent salient concepts, and then applying or creating a framework to organize the data in a way that can be used to describe or explain a phenomenon [7]. We worked as a team to develop a common understanding of the scope of analysis and agreed on key terms to search for in these documents (adolescent, gender, multi-sectoral and community). Given the length of these original source documents, any text that was relevant to these terms was copied into word documents, which were then referred to for further content analysis. We piloted and then applied a questionnaire for data extraction to support country-level analyses.

Our analysis followed a phased approach to understanding how adolescent health, particularly their sexual and reproductive health, is addressed by the GFF. We first foregrounded our analysis by collating secondary data that provided contextual information on adolescents across all the countries examined. This enabled us to outline the social and health needs of adolescents that should be addressed by these planning documents.

We secondly examined the extent to which adolescent health is acknowledged in the planning documents in terms of programming content, indicators and investment. To further synthesise our findings, we created a scoring table that categorised a country document as red if it had no mention of adolescents in it, orange for minimal reference to adolescents and green for more than a minimal reference. Further information supporting the scoring of each element across the documents is listed in Additional file 1.

Moving beyond a descriptive summary of whether adolescents are mentioned or not, we then delved further into the definitions used for adolescents, the rationale for working with them and the range of adolescent health conditions covered in addition to sexual and reproductive health. Given that adolescence, including adolescent health and adolescent sexual and reproductive health, is understood in different and partial ways by varied constituencies, this initial analysis is an important step in creating a stronger common foundation from which to address this population.

While a key interest is adolescent sexual and reproductive health, given the evolving understanding of adolescence and adolescent health, we did not compartmentalize it from its broader social determinants or health systems in which they are embedded. Our analytical approach therefore considered three different framings or lenses: a service delivery lens focussing on tangible inputs for programs; a societal lens highlighting the implicit and explicit social relationships involved; and a systems lens which emphasises change dynamics, including by those actors outside of the health care sector [8]. These lenses reflect developments in health policy and systems thinking that acknowledge technocratic programmatic inputs, but also enable consideration of more complex social realities and health systems actors. The three lenses are complimentary and together provide a more comprehensive framework to support health reforms.

An analysis workshop from August 21 to 22, 2018 used the country-level analysis to further synthesise the information across countries comparatively. The presentation was shared at global conferences through side events organised by Countdown 2030 in 2018 and 2019, as well as proactively shared with UN agencies and with the GFF Secretariat. Further follow up correspondence and a meeting with the GFF secretariat elicited their feedback in 2019, prior to drafting the submission. The GFF Secretariat did not play any role in influencing our research protocol or analyses/framing of research findings.

The study was exempt from ethical review since it is not human subjects research. The documents which formed the data source for the analysis are publicly available, and therefore no special permissions were required.

## Results

We first present the context of adolescents in the countries we included in our analysis to understand the social and health characteristics that shape their health, sexuality and reproduction. We then descriptively assess whether the planning documents mention adolescents in terms of programming content, indicators and investment. We review how adolescents, their health and sexual and reproductive health is mentioned, before analysing the content as per the service delivery lens (range of programmatic services), the societal lens (vulnerabilities, rights, gender and male engagement reflecting key social relationships), and the systems lens (multi-sectoral, community engagement, adolescent engagement as elements of change dynamics by actors outside of health).

### Social and health contexts of adolescence in study countries

Adolescents make up a fifth to a quarter of the total population across all the study countries (Table 1), making them a significant demographic priority. Secondary school completion rates are lower for adolescent girls than for boys across all the study countries, with the percentages being extremely low in Tanzania and Mozambique for both girls and boys. Adolescent girls are also vulnerable as substantial percentages of adolescent girls are married or in union before the age of 18 (ranging from 23% in Kenya to 59% in Bangladesh), with significant percentages married or in union by age 15 (18% in Nigeria, 22% in Bangladesh).

**Table 1:** Demographic context of adolescents in study countries

First wave countries	% of population 10–19	% female secondary school completion	% male secondary school completion	% Women married/in union by age 15	% Women married/in union by age 18
Bangladesh	20	26	31	22	59
Cameroon	23	12	18	10	31
DRC	23	21	30	10	37
Ethiopia	25	12	13	14	40
Guatemala	23	–	–	6	30
Kenya	23	38	44	4	23
Liberia	23	9	18	9	36
Mozambique	24	4	8	14	48
Nigeria	23	42	57	18	44
Uganda	25	13	18	10	40
Tanzania	23	2	4	5	31
	(2016)	(2016)	(2016)	(2010–2017)	(2010–2017)

Data sources

% of population 10–19: United Nations Population Division population.un.org/wpp/ (Accessed 25 Sept 2018)

Secondary school completion rate: United Nations Children’s Fund Global databases data.unicef.org/topic/education/overview/ (Accessed 25 Sept 2018)

% women married/in union by age 15 or 18: United Nations Children’s Fund Global databases data.unicef.org (Accessed 25 Sept 2018)

In terms of health outcomes (Table 2), HIV incidence estimates vary considerably across the study countries, revealing how important it is to not make generalisations across low and middle income countries or even sub-Saharan Africa. It is striking that 20–40% of adolescent girls give birth before turning 18 years of age. Despite the vulnerability of adolescent girls to social determinants that undermine their sexual and reproductive health in particular, it is important to also note that adolescent boys suffer higher mortality across almost all study countries, particularly in Guatemala.

**Table 2:** Health context of adolescents in study countries

First wave countries	HIV incidence per 1000 uninfected population – age 15–19	% of women birth before age 18 2011–2016	Female age 10–19 mortality rate (per 100,000)	Male age 10–19 mortality rate (per 100,000)
Bangladesh	< 0.01	36	56	72
Cameroon	2.4	28	254	292
DRC	0.24	27	238	266
Ethiopia	0.23	22	163	220
Guatemala	0.1	20	68	128
Kenya	2.69	23	180	234
Liberia	1.49	37	185	215
Mozambique	3.03	40	272	286
Nigeria	2.18	29	340	340
Uganda	2.55	33	203	243
Tanzania	1.04	22	202	254
	(2016)	(2011–2016)	(2015)	(2015)

*Data sources* HIV incidence per 1000 population: UNICEF. 2017. State of the World's Children 2017. Geneva: UNICEF

http://data.unicef.org/resources/state-worlds-children-2017-statistical-tables/ (Accessed 25 Sept 2018)

% women birth before age 18: UNICEF. 2017. State of the World's Children 2017. Geneva: UNICEF

http://data.unicef.org/resources/state-worlds-children-2017-statistical-tables/ (Accessed 25 Sept 2018)

Adolescent mortality rate: World Health Organization, Global Mortality Database www.who.int/healthinfo/mortality_data/en/ (Accessed 25 Sept 2018)

In reviewing access to sexual and reproductive health for adolescents (Table 3), the percentage of adolescents girls aged 15–19 with demand for family planning satisfied with modern methods is overall quite low, but varies significantly, ranging from 19% in DRC to 61% in Ethiopia. Access to services for sexual and reproductive health is further restricted due to restrictions of the rights involved, particularly for adolescent girls. In DRC, Liberia and Mozambique, adolescent girls cannot access family planning without consent from either their parents or spouses. With regards to access to abortion, it is largely only available to save a woman’s life or for her health. While globally 30% of adolescent girls aged 15–19 experience physical and/or sexual violence by an intimate partner [9], only Cameroon, Ethiopia and Cameroon allow abortion in cases of rape or incest.

**Table 3:** Context of adolescent sexual and reproductive health across study countries

First wave countries	% women FP modern methods satisfied - age 15–19	without spousal or parental consent	Legal status of abortion
Bangladesh	47	no data	Partial (1)
Cameroon	53	no data	Partial (1,2,3, 6)
DRC	19	No	Partial (1)
Ethiopia	61	Yes	Partial (1, 2, 3, 6, 7)
Guatemala	48	Yes	Partial (1)
Kenya		Yes	Partial (1, 2, 3)
Liberia	36	No	Partial (1, 2, 3)
Mozambique	31	No	Partial (1, 2, 3, 6, 7)
Nigeria	28	Yes	Partial (1, 2, 3)
Uganda	44	Yes	Partial (1, 2, 3)
United Republic of Tanzania	41	Yes	Partial (1, 2, 3)
	(2011–2016)	(2013–2016)	(2015)

Data sources

% women age 15–17 FP modern methods satisfied: Countdown to 2030 compiled data from household surveys (DHS and MICS)

Adolescent family planning without spousal or parental consent: Countdown to 2030 compiled data from WHO-MNCAH Policy Indicator Database http://www.who.int/maternal_child_adolescent/epidemiology/policy-indicators/en/ (Accessed 25 Sept 2018)

Legal status of abortion (1) To save a women's life, (2) to preserve physical health, (3) to preserve mental health, (4) for economic & social reasons, (5) on request, (6) in case of rape or incest, (7) in case of foetal impairment: Countdown to 2030 compiled data from WHO-MNCAH Policy Indicator Database http://www.who.int/maternal_child_adolescent/epidemiology/policy-indicators/en/ (Accessed 25 Sept 2018)

### Are adolescents in the GFF?

In this section, we assess the extent to which adolescents and their health is mentioned in the planning documents in terms of programming content, indicators and investment. With an aim to encouraging a common foundation from which to address adolescent health, we also review how the planning documents refer to adolescents, what age ranges are specified and what health conditions are detailed.

Adolescents are generally included as part of the broader RMNCAH-N acronym in the GFF country planning documents. Standalone sections do exist, varying from a single paragraph to more extensive detail across the documents. Despite good examples across the country planning documents, there is a dilution of attention to adolescents as we move from programming content to indicators to actual investments across both Investment Cases and Project Appraisal Documents (Table 4). Even when investments specific to adolescents are detailed, they can be minimal given the overall funding envelope.

**Table 4:**
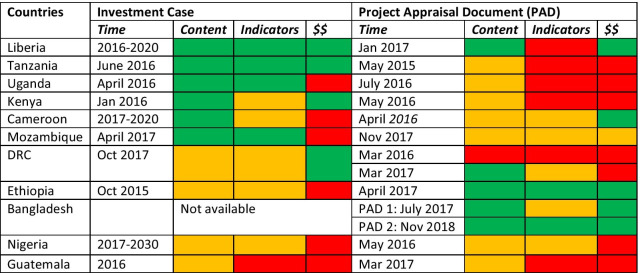
Extent to which attention is paid to adolescents in the Global Financing Facility country planning documents

Key: red for zero, orange for minimal, green for more than minimal (summary information for each ranking are in Additional file 1)

While most Investment Cases were followed by Project Appraisal Documents chronologically, the linkages between planning undertaken in the Investment Cases and commitments in the Project Appraisal Documents are largely not discernible from examining these documents. While the Liberia Investment Case and Project Appraisal Documents were largely aligned in responding to adolescents, it was the only one to do so. Tanzania, Uganda and Kenya had strong inclusion of adolescent health in their Investment Cases, but this did not turn into commitments in their Project Appraisal Documents. In contrast, the Project Appraisal Document was an improvement from the Investment Case in terms of addressing adolescents in Ethiopia. For the remaining countries, there was little overall difference in terms of addressing adolescents between these planning documents, for better or worse.

When there is any analysis of adolescents in these first GFF country documents, it is largely as a population made vulnerable due to a lack of services and the social determinants that place them at risk. In half the country documents, they are also mentioned as an important demographic group with key economic dividends for future development. None of the country documents mention that adolescence is an important developmental phase in its own right. The issue of adolescent rights, particularly in the context of sexuality and reproduction, was mentioned in the Ugandan, Kenyan, Nigerian, Liberian and Bangladesh documents (box 1). However, how this was linked to investment was unclear.

**Table:** Box 1: Mention of adolescent rights in GFF country documents for first 11 countries

Uganda clearly discusses adolescent rights, empowerment, voice/participation	
Liberia flags empowering adolescents and securing adolescents’ rights to health through strengthening laws against early marriages, domestic violence and harmful practices	
Kenya lists legal and rights frameworks and acknowledges these as not recognised enough	
Nigeria lists Child Rights Act, Violence against Person’s Prohibition Act, National Commission of Women Act	
Bangladesh notes “Women and girls in Bangladesh face various barriers and impediments that make it difficult if not impossible for sexual and reproductive health rights to be realized…There is no single policy or strategy document issued by the government on sexual and reproductive health rights.”	

Across these country documents, the age range or definition of adolescents does not necessarily align with WHO norms, making comparisons challenging at times. Overall adolescents are referred to as a homogenous group and also heteronormatively with some acknowledgement of specific vulnerabilities often in a situation analysis section. Documents for Ethiopia, Kenya, Cameroon, DRC and Uganda mention particular social contexts that heighten the vulnerability of adolescents whether with regards to location (urban/rural divides, homelessness, incarceration, out of school) or with regards to well-being (living with disability or HIV without a supportive environment). Documents for Ethiopia and Nigeria also differentiated adolescent health needs within conflict settings, with Nigerian documents flagging the need for counselling in instances of sexual assault. In contrast, there was no mention of how conflict settings increase vulnerability for adolescents in the documents for Cameroon, DRC, Uganda and Liberia.

When examining the range of adolescent sexual and reproductive health conditions and needs covered by these GFF country documents, most of them mentioned teenage pregnancy as a priority. The documents for Kenya mentioned that although its total fertility rate has declined, its teenage pregnancy rate has not. In the Nigerian documents, it was noted that the median age at first birth has remained at 20 for many years. When specified, access to family planning was noted as critical for adolescents, and addressing/preventing/delaying early marriage being a priority for adolescent programming in Mozambique, Cameroon, Kenya, Ethiopia, Uganda and Liberia.

Other adolescent health conditions, like mental health and substance abuse, are acknowledged, but without corresponding interventions or programming content. Adolescent nutrition is mentioned in several country documents, but without great depth, even in the Guatemalan documents, which focussed on chronic malnutrition. The Cameroon and Uganda documents are clear outliers in discussing a holistic approach to adolescent health and a comprehensive listing of health conditions, with the Ugandan Investment Case recommending a broad set of packages for investment as well.

### Service delivery lens: programmatic entry points for adolescent sexual and reproductive health services

In looking at what services were supported for adolescent sexual and reproductive health, several countries mentioned two main approaches: adolescent friendly health services and school health programs; although these were not systematically mentioned or invested in across all country documents.

Adolescent friendly health services were featured as part of the service package or assessed through an indicator in documents for Ethiopia, Kenya, Tanzania, Liberia, Mozambique and Uganda. In documents for DRC, while the term adolescent friendly services was not mentioned, health worker training and reducing stigma faced by adolescents was seen as key in removing barriers to access services among this key population. In documents for Mozambique, the increase in use of Adolescent and Youth Friendly Health Services has not been able to keep up with demand, even as data reveal that many adolescents are not aware of their existence. In the documents for Nigeria, while adolescent friendly services were mentioned this was not connected to programmatic investment.

For Ethiopia, Kenya, Liberia, Mozambique, and Uganda, comprehensive sexual education (CSE) is part of the school health program supported by GFF country documents. In Bangladesh, the GFF includes a Project Appraisal Document dedicated to secondary education that scales up school-based health programming nationwide, including support for an Adolescent Girl’s Program (Box 2), alongside training secondary and madrasah teachers on essential life skills for young girls, complimented with nation-wide awareness campaigns.

**Table:** Box 2: Bangladesh Adolescent Girl’s Program in Schools

Key features include (a) incentives to female students in grades 9–12 from economically disadvantaged areas; (b) separate functional toilets for girls to reach a national minimum standard ratio as specified in the operations manual; (c) inclusion of relevant adolescent health topics in curriculum including sexual and reproductive health, gender equity, good nutrition and staying fit; (d) promotion of menstrual hygiene with disposal facilities in schools and at home; (e) promoting positive student relationships and tackling bullying and gender-based victimization; (f) inclusion of adolescent health in teachers’ ongoing professional development; (g) awareness raising around adolescent health and health services for students, teachers, and community; (h) formation of school-based girls committees supported by female guardian teacher; (i) introduction of student and peer counseling; and (j) initiating nutrition services for girl students to address underweight and anemia; and (k) promoting links between schools and local health services	

### Societal lens: key social determinants framing adolescent sexual and reproductive health

For this lens, we focussed on gender as a key social determinant of adolescent sexual and reproductive health. Almost all the country documents acknowledged gender inequality as a key driver undermining adolescent health. Gender norms, bias and the low status of women and girls were noted as problems in documents for Tanzania, Kenya, and DRC, but no corresponding programmatic interventions or recommendations were made. In documents for Liberia and Ethiopia, the National Gender Policy was referred to, and the importance of women’s empowerment noted, but no recommendations or investments made specifically.

Mozambique stands out as having gender content included across its planning documents. The Investment Case notes the Ministry of Health’s new Strategy for the Inclusion of Gender in the Health Sector, and its collaboration with the Ministry of Gender, Children and Social Action, the Ministry of Education and Human Development. The Project Appraisal Document flags gender as a cross‐cutting consideration, in terms of analysis, target groups, and specific interventions to address social norms and inequalities. These include community‐based interventions to engage men in family planning and sexual and reproductive health activities; that gender‐based violence is reflected in the curriculum of health professionals, including at community level; and that gender and socio‐cultural sensitivity and gender responsiveness access dimensions are included in health facility scorecard and community consultations. In documents for Nigeria and Bangladesh, the need for gender sensitive health systems and planning was also mentioned, with the importance of sex-disaggregated data, community and male engagement noted, gender responsive checklists for health facilities, gender based violence training for health providers and gender balanced human resources.

The Bangladesh Education Project Appraisal Document invests in an Adolescent Girl’s Program, which addresses retention in schools, but has gender and health components strongly integrated into it (Box 2).

In documents for Liberia and Bangladesh, both girls and boys are mentioned as key populations for school programs and health education. In the remaining documents, while adolescent girls were the focus of much of the analysis, adolescent boys were mentioned minimally, about twice across each of the documents, mostly through disaggregated statistics. In documents for Nigeria, there was more discussion about boys given that men and boys were also threatened by Boko Haram and vigilante groups. Male engagement, was listed in the documents for Tanzania, Mozambique, Nigeria, Uganda and Cameroon, mainly as a means to support women’s access to services, rather than as a means to transform gender norms and power relations. There was no mention of adolescent boy’s in the Guatemala documents, which focussed primarily on chronic malnutrition, despite the high levels of mortality experienced by adolescent boys there.

Gender based violence was also mentioned as an important area for intervention in several country documents. While this was not always linked to adolescents, there were important exceptions. In documents for Liberia, Uganda and Kenya, gender based violence interventions specifically named adolescents as a key group to address. In documents for Ethiopia, this was noted as a separate strategy led by the Gender Ministry. In contrast, documents for DRC discussed sexual violence without linking it to adolescents. In documents for Tanzania, while it was noted that adolescent girls were twice as likely to experience gender based violence than adolescent boys (24% vs. 13%), no interventions were recommended.

### Systems lens: change agents catalysing adolescent sexual and reproductive health

From a systems lens, we examined the multiple actors that contribute to health beyond the health care sector and across health system levels that can support positive change for adolescent health. Several countries mentioned the importance of multi-sectoral action and list a range of development sectors to be involved, but usually without concrete investments, processes, focal points or indicators to ensure that action follows. Two exceptions were the planning documents for Cameroon and Liberia which specified multi-sectoral coordinating bodies at national, district and municipal/ county level to support implementation. Liberia furthermore mentioned establishing robust feedback systems and mechanisms through quarterly stakeholder fora, and other de-concentrated forms of governance and mechanisms for inter-sectoral dialogue.

Positive examples of multi-sectoral investments found in the documents from Kenya and Cameroon include conditional cash transfers to keep girls in school. The Kenyan documents also listed income generation measures to support the socio-economic needs of adolescents not accepted by their parents. As mentioned earlier, Bangladesh, through its Education Project Appraisal Document, supported an adolescent girls program with incentives to complete school, attention to toilets in schools and menstrual hygiene, curriculum reform, addressing bullying and gender victimisation, as well as setting up girls committees.

As mentioned earlier, a large proportion of adolescents are not in school or accessing health services across the countries examined (Tables 1, 2). Only the Cameroon and Liberia documents addressed this explicitly by supporting youth centres and girls clubs outside of schools. In addition, the Uganda documents listed community awareness raising days or forums with adolescents, the Liberia documents support peer-to-peer education through community pregnancy prevention advocacy groups and the documents from Mozambique stress community outreach programs for adolescents. Documents from Cameroon, DRC, Liberia, Mozambique and Uganda specifically mentioned adolescents as a group for community health workers to work with.

In the documents for Tanzania and Kenya, community and local government authorities were recognised as key actors for supporting access to sexual and reproductive health information and services, as well as representation in local planning. However, neither of these areas were allocated budgets for follow up in the documents. In Mozambique, there was further investment in supporting the mobilisation of community and religious leaders, particularly in disseminating awareness of legislation against early marriage.

Family members were also recognised as a key group to support for adolescent health, with reference to godmothers and godfathers in Mozambique and mother-in-laws and parent groups in Tanzania, Cameroon and Uganda. In Tanzania, the National Youth Adolescent Parent Community Alliance (NYAPCA) was supported to provide clinical and non-clinical SRH services, as well as recreational activities, small library/learning services, and livelihood activities.

While the role of other development sectors and actors was acknowledged, across all the documents, we found no mention of commercial determinants of adolescent health. For example, while substance abuse was mentioned, the specific role of smoking and alcohol as a detrimental influence on adolescent health was not mentioned in any of the country documents. The rise in obesity and diabetes among the young is a consequence of how highly processed and cheap food and sugar driven by multinational corporations have increasingly become the norm.

Across several country documents, engagement with adolescents themselves as a key constituency was recognised as important, but largely an area that was noted to be weak and where future work needed to be done. Only the Mozambique Project Appraisal Document noted consultations with adolescent girls. Documents from Uganda and Mozambique acknowledged access to data as empowering and the potential of digital communication for health promotion and peer support networks, but without concrete investment linked to adolescent engagement. The Bangladesh education-related PAD specifies the formation of school-based girls committees supported by female guardian teachers. However, it is not clear what these committees will focus on, and if they have any involvement in planning, design, implementation or monitoring of the Adolescent Girl’s Program. In contrast, the Liberian documents supported 100 National Youth Volunteers to monitor and report on reproductive health commodity stock levels at targeted health facilities to inform forecasting, quantification and distribution of commodities to adolescents and young people. These same youth volunteers are to supervise youth related programming for adolescent sexual and reproductive health and link with activities supported by the Ministry of Youth and Sports.

## Discussion

Adolescents are recognised by the GFF leadership as a key priority [1] and several positive examples of corresponding understanding and investment were found. While this is a good start, our analysis of GFF investment documents in their first eleven countries highlight that much more could be done to consistently address adolescent needs and determinants of health, and their sexual and reproductive health in particular, as a strategic national development priority. The dilution of attention as one moves from programming content, to indicators to investment is concerning, as is the lack of a common approach to defining this key population, consistently acknowledging and supporting their health needs and rights, and investing in the social determinants that underpin their health. This is particularly critical given forthcoming waves of replenishment financing.

Adolescents are a substantial and critical population across all the study countries. The secondary data highlight the complexity of addressing adolescents given that many of them are out of school and substantial proportions of adolescent girls are married. These social determinants shape their sexual and reproductive health needs, which vary by geography, gender, life stage etc. As a result, access to sexual and reproductive health services for adolescents is marked by multiple barriers. For these reasons more consistent investment in the recognised programmatic entry points for health are needed, alongside concurrent investment in addressing gender among other social determinants and engaging with actors beyond the health sector.

Our analysis highlights several areas requiring further support. Programmatically, while a strong focus on teenage pregnancy was found, more comprehensive approaches to other aspects of sexual and reproductive health and to other adolescent health issues are needed. The Lancet Commission notes that while progress on adolescent sexual and reproductive health has been made, many countries have multiple adolescent morbidities, with adolescent health conditions related to injuries and violence, nutrition and mental health remaining neglected [3, 10]. Even when considering the unfinished agenda in adolescent sexual and reproductive health, more consistent disaggregation by age and sex, and attention to early adolescence is needed [11].

While a range of social determinants that are particularly challenging for adolescents were noted in some of the documents, more must be done to acknowledge and address the needs of highly marginalised adolescents, such as LGTBQI adolescents. Furthermore, given that increasing numbers of the most marginalised populations are caught in humanitarian settings, more consistent attention to the ramifications of conflict settings for adolescent health is critical. The lack of focus on adolescents in GFF documents for study countries that are either experiencing humanitarian crises or hosting crises-affected populations is concerning, and demonstrates that young people, including adolescents, continue to be a neglected group in humanitarian settings [12].

From a service delivery lens, much more depth and breadth is needed in strengthening adolescent and youth friendly health services and programs. Progress has been made in terms of moving beyond previously piecemeal and ineffective approaches that included stand-alone adolescent health services or one off training for health workers. A number of countries have developed national standards for adolescent and youth friendly services and included them in pre-service training for health workers [13]. Efforts to strengthen implementation at scale without compromising on quality or equity are critical, with attention to varying social contexts across subnational levels [11].

The education sector is particularly important for shaping adolescent behaviour, health and well-being [3, 14, 15]. School-based health interventions are popular with young people [16] and provide important mental and reproductive health services [17, 18]. Sexual and reproductive health education, counselling, and contraceptive provision are effective in increasing sexual knowledge, contraceptive use, and decreasing adolescent pregnancy [19]. A comprehensive approach also addresses issues of sexuality, sexual orientation and gender identity, power relations and harmful constructions of masculinities and femininities. Partnerships with teachers, administrators, parents and local leaders are key to ensure that conservative pushback and reticence about providing comprehensive sexual education and contraceptives do not undermine programs that are often under-resourced and poorly supported [13].

While attention has primarily focussed on the direct impacts from health education and services, the importance of the overall school environment or ethos for supporting adolescent well-being and retention for health in the long run must not be neglected [3]. Indeed, the benefit to health from the core business of education for adolescents is greater than from health services delivered using the educational system as a platform. A genuinely multisectoral investment case for adolescents must prioritize the coverage and quality of education, and engage with the education sector as a key stakeholder. By contrast, the GFF process, with the exception of Bangladesh, remains mostly focused on the health sector.

From a societal lens, gender inequality as an intersectional and structural driver of adolescent health [20] must be further addressed for the GFF investments to be socially relevant, transformative and effective. It is the key social determinant underlying adolescent pregnancy, vulnerability to HIV and STIs, violence against adolescent girls and women, female genital mutilation and stigma related to menstruation [21, 22]. It also strongly influences adolescent mental health, substance use, road traffic injuries and other risks. While adolescent girls are more vulnerable with regards to the drivers, incidence and consequences of sexual and reproductive health, adolescent boys cannot be entirely ignored [23, 24] given their own gendered vulnerabilities and roles in advancing gender equality. Adolescence is a critical period for developing autonomous and critical thinking [3, 14, 21]. Successful programs that are participatory in nature [25] have built self-esteem, negotiating skills and agency in girls, while supporting boys to recognise their own privileged status and reward them when challenging conservative gender norms rather than reinforcing them [21]. Following a socio-ecological model, supportive measures must also involve parents, peers, schools, community structures and social media [3, 14, 24].

From a broader systems level, several opportunities are missed if multi-sectoral and community initiatives are not themselves leveraged for adolescent health. This is particularly critical given that large proportions of vulnerable adolescents are not in school. Community level social institutions, such as church groups, neighbourhood health committees, youth groups must be engaged, alongside community health workers [26]. Addressing the ingrained social norms, gender inequality and poverty that underlies the persistence of child marriage, female genital mutilation, and gender based violence requires both widespread community mobilisation, as well as structural interventions involving cash transfers, women’s rights to property, and corresponding legal and policy reforms [13, 19].

Systems level change cannot be undertaken without critically engaging with adolescents and young people themselves. Meaningful involvement of adolescents in leadership and participation during design, implementation, monitoring and evaluation contributes to the accessibility, acceptability, quality and outcomes of programmes [3, 14, 27]. Increasingly normative guidance recommends adolescent engagement and some key donor require it [13]. Their mobilisation and political activism has been essential in securing national legislative rights and international commitments [3, 13].

The recommended program areas and interventions mentioned above, while not consistently funded through the Project Appraisal Documents, could be funded by other World Bank agreements and donors. However, the process for ensuring corresponding investments from other World Bank agreements and donors remains unclear from the planning documents accessed. More should be done by the GFF Secretariat and country partnerships to track this on the GFF website. Concerns have been expressed about capacity at country level to support the GFF process and its alignments with national planning processes [28, 29]. Country planners may focus on the World Bank’s commitments concretised in the Project Appraisal Document and neglect other stakeholders. Other donors may also not agree with the processes facilitated by the the GFF Secretariat and country partnershipsBank [2, 30]. There are minimum standards related to inclusiveness and transparency at country level, but this does not ensure civil society participation in decision-making processes [31]. These country level dynamics call into question how effective Investment Cases are in ensuring actual investments [31] and the importance of focussing on the development of the Project Appraisal Documents as a key process through which commitments are made.

Finally, while domestic resource mobilisation is on the rise, it remains woefully inadequate to support even the basic service package in low income countries [32]. Whether the GFF process will actually translate into increased domestic resource mobilisation or a substitution across different sectors remains to be seen [29].

The GFF approach aims to be fully embedded in country planning and programming cycles, especially the overall health sector strategic plans and the associated RMCNAH-N program plans. If prioritised RMNACH-N interventions within the country planning process are funded, the GFF could result in significant health gains [33]. Current modelled estimates of lives saved do not yet consider the impact of adolescent health interventions and thus may underestimate their true impact [13, 34]. However, the challenges to ensuring consistent attention to adolescent health in the GFF country planning process are not technical alone.

The GFF process is contingent upon the details of operationalisation at country level [28] and is also political in nature. The extent to which countries prioritize adolescent health, including sexual and reproductive health, is likely to have major influence on the ultimate priorities and investments made. Adolescent sexual and reproductive health remains a highly contested area where adolescent rights, agency and autonomy are in tension with conservative social norms and political forces enforcing such norms [3, 13]. It is also an area where the social determinants of health and the engagement of sectors outside of health is critical. Further efforts in creating a common foundation for understanding adolescent sexual and reproductive health and clarity on the theory of change for adolescent health more broadly in the GFF is required. Strong leadership, technical and advocacy support from the GFF is required to attain greater and more systematic attention for adolescent health without undermining country leadership and ownership in the process.

Our analysis of how well the GFF systematically addresses adolescent health, and adolescent sexual and reproductive health in particular, has its strengths and weaknesses. The analysis is restricted to the data sources that are publicly available: the Investment Cases and the Project Appraisal Documents at the time of analysis. These documents represent one measure of priorities and commitments by revealing whether and how adolescents are acknowledged, what is programmatically focussed on, what is measured and what resources are prioritised. While we sought feedback from global actors on our analysis, and several co-authors have worked, or continue to work on GFF processes for global agencies, our analysis is preliminary and primarily undertaken by health policy and systems researchers engaged with RMNCAH-N based outside the World Bank. The number of cases are too small to generalise for specific regions. Furthermore, our analysis can only reveal what is on paper, not what is practiced, nor what was intended by the authors of these documents. Nor does a content analysis reveal the power dynamics involved in terms of the negotiations brokered, the actors engaged or ignored, or the actual implementation that followed these documents. Nonetheless, these planning documents and the priorities they reflect are a first part of descriptive research that lays the foundation for further independent research on how the GFF is impacting RMNCAH-N and health systems.

## Conclusions

Adolescent health is noted by GFF leadership as a key priority, and several country planning documents signal this recognition. Rather than see the glass as half-full and assume that progress is inherent, more could be done to ensure that adolescents are consistently addressed within GFF planning documents, given the missed opportunities detailed in our analysis. To seize the opportunity to lead on adolescent health, and adolescent sexual and reproductive health in particular, for the thrive and transform agenda of the Every Woman and Every Child Global Strategy, more of the same is neither enough nor relevant.

Apart from working with countries to adopt the WHO definition of adolescents and youth, the GFF should also more consistently support prioritisation and contextualisation of adolescent health based on contextual analysis supported by existing secondary data.

Further deepening and widening of service delivery approaches by the GFF in including adolescent and youth friendly health services and school based programming is required. Critically, adolescent sexual and reproductive health requires approaches that go beyond traditional health service approaches as important as they are. In addition, addressing gender power relations through approaches that are multi-component and multi-level are required. These types of investments are not low-hanging fruit, but are nonetheless strategic in sustaining gains in outcomes over the longer term. The GFF must further invest in governance approaches that facilitate multi-sectoral collaboration, community engagement and youth involvement are critical, given that the actors and sectors that influence adolescent health sit outside the health sector.

This requires capacity building by the GFF to ensure utilisation of the evidence base underpinning transformative adolescent health investments, but also efforts made in canvassing constituencies, including adolescents themselves, to shore up political capital. Although much progress has been made on adolescent sexual and reproductive health, there remains pushback and discomfort regarding several key components, whether sexuality education, safe abortion or access to contraceptives. Key allies for adolescent health within education, social protection and employment sectors must not remain sidelined. The GFF, despite its own constraints, could further advance investments in adolescent sexual and reproductive health, but only if it addresses adolescent health consistently through all its country planning processes, and, moreover, is able to bring together all stakeholders and navigate the governance required to transform its theory of change into practice.

## Supplementary Information

**Additional file 1.** **Supplementary file 1:** Summary sentences behind scoring the extent to which attention is paid to adolescents in the first 11 countries involved in the Global Financing Facility.

## Data Availability

Data sharing is not applicable to this article as no datasets were generated or analysed during the current study. Data and materials used for this article are publically available through the GFF website.

## Abbreviations

GFF: Global Financing Facility
IDA: International Development Association
IBRD: International Bank for Reconstruction and Development
RMNCAH-N: Reproductive, maternal, newborn, child, adolescent health and nutrition
