# Supplementary material for: Are rhetorical commitments to adolescents reflected in planning documents? An exploratory content analysis of adolescent sexual and reproductive health in Global Financing Facility country plans
Source: Reprod Health. 2021 Jun 17;18(Suppl 1):124. doi: 10.1186/s12978-021-01121-y (PMC8210342; doi:10.1186/s12978-021-01121-y)
Supplement: Supplementary file 1 — Additional file 1. Supplementary file 1: Summary sentences behind scoring the extent to which attention is paid to adolescents in the first 11 countries involved in the Global Financing Facility. [file 12978_2021_1121_MOESM1_ESM.docx]

Supplementary file 1: Summary sentences behind scoring the extent to which attention is paid to adolescents in the first 11 countries involved in the Global Financing Facility

| **Countries** | **Investment Case** | | | | **Project Appraisal Document (PAD)** | | | |
| --- | --- | --- | --- | --- | --- | --- | --- | --- |
|  | ***Time*** | ***Content*** | ***Indicators*** | ***$$*** | ***Time*** | ***Content*** | ***Indicators*** | ***$$*** |
| Liberia | 2016-2020 |  |  |  | Jan 2017 |  |  |  |
| Tanzania | June 2016 |  |  |  | May 2015 |  |  |  |
| Uganda | April 2016 |  |  |  | July 2016 |  |  |  |
| Kenya | Jan 2016 |  |  |  | May 2016 |  |  |  |
| Cameroon | 2017-2020 |  |  |  | April *2016* |  |  |  |
| Mozambique | April 2017 |  |  |  | Nov 2017 |  |  |  |
| DRC | Oct 2017 |  |  |  | Mar 2016 |  |  |  |
|  |  |  |  |  | Mar 2017 |  |  |  |
| Ethiopia | Oct 2015 |  |  |  | April 2017 |  |  |  |
| Bangladesh |  | Not available | | | PAD 1: July 2017 |  |  |  |
|  |  |  |  |  | PAD 2: Nov 2018 |  |  |  |
| Nigeria | 2017-2030 |  |  |  | May 2016 |  |  |  |
| Guatemala | 2016 |  |  |  | Mar 2017 |  |  |  |

Key: red for zero, orange for minimal, green for more than minimal, grey not available

Liberia

- Investment case
  - Content: Adolescent health included as one of six priority areas with broad range of issues raised (including FGM, GBV) and solutions presented.
  - Indicators: Results framework includes target for adolescent birth rate – from 31% in 2013 to 25% in 2021. It also includes comprehensive list of activities to undertake relating to all priority areas in the investment case that will be reviewed quarterly.
  - Investment: There is an investment included for adolescent health of nearly USD 12m over five years (for programming 6.7 million and operational research 5.2 million).
- Project Appraisal Document:
  - Content: Included as one of six priority areas with broad range of issues raised
  - Indicators: None, although they will consider performance based indicators related to adolescent health in the review of PBF indicators to be approved.
  - Investment: Adolescent health is listed as a sub-component of strengthening quality of care at PBF facilities. Specific amount allocated for this is US$1m additional resources.

Tanzania

- Investment case
  - Content: : Adolescent health mainly focuses on health sector with some mentions of drivers, and features in the M&E framework
  - Indicators: Mainly SRH and HIV-focused indicators that are adolescent-specific, with one indicator on proportion of 15-19 year olds married/cohabiting
  - Investment: Adolescent health-focused activities are costed at $57,472,939 USD (Key Result Area (KRA) 6.3 for Adolescent reproductive health costed at $57,315,872 USD; and KRA 6.5.3 on HPV vaccination coverage among adolescent girls costed at $157,067), which amounts to 4.3% of the overall funding envelope. It should also be noted that integrated training and guidance documents for maternal, newborn, child and adolescent health are also costed in KRA 6.1.9 ($3,582,629) and KRA 6.1.11 ($384,549).
- Project Appraisal Document:
  - Content: Adolescents are only mentioned sparsely, where they are embedded within the “A” in the “RMNCAH” acronym, and included as part of the definition of “vulnerable” populations. However, adolescents are not focused on as an individual target group.
  - Indicators: None
  - Investment: None

Uganda

- Investment case
  - Content: Adolescent health is mentioned comprehensively including its multi-sectoral determinants
  - Indicators: Outcome indicators included for teen pregnancy and child marriage with broad drivers as key results in framework.
  - Investment: No specific mention of investment (allocations/ Disbursement) for any topic
- Project Appraisal Document:
  - Content: Adolescent health mentioned as part of RMNCAH with emphasis on adolescent friendly health services
  - Indicators: None
  - Investment: None

Kenya

- Investment case
  - Content: Adolescents are acknowledged as an important part of RMNCAH with corresponding interventions detailed. Adolescent health is framed broader than SRH to include, mental health, substance abuse etc. but this is not clearly evident in programming and M&E.
  - Indicators: Results Framework only has teenage birth rate and female genital mutilation for 15-19 years that is adolescent specific. For most other indicators it refers to women 15-49 years with a SRH focus.
  - Investment: Limited allocation to adolescent health with reference of resource requirements for Nation-wide Scale-up where adolescent and youth get allocated 5% of total. Adolescent are ‘subsumed’ in other categories such as family planning and maternal and newborn health.
- Project Appraisal Document:
  - Content: Acknowledgement that multi-sectoral collaboration is important for adolescent health. Very little mention of adolescents and only within the RMNCAH acronym, which is very different from the investment case.
  - Indicators: No adolescent specific indicators in Results Framework, and they are subsumed under maternal and reproductive indicators
  - Investment: PAD is titled Transforming health systems for universal care project and not evident what and how resources will be allocated to adolescent health. Mention of resources from other donors which focus on HIV/AIDS, nutrition, GBV, Human rights, malaria etc.

Cameroon

- Investment case
  - Content: Sexual and reproductive health of adolescents discussed at length based on a holistic framework considering school retention, life skills and socio-economic needs.
  - Indicators: Many proposed indicators related to adolescent health listed in annexes, but whether they will be used is unclear
  - Investment: Little detail about any specific budget in the investment case
- PAD:
  - Content: Adolescent health not discussed as much as in the investment case
  - Indicators: One intermediate results indicator: Adolescent girls aged 10-19 years benefiting of multisectoral services supported by the GFF IC (number).
  - Investment: 5.5% of funding (IDA + GFF = $127 million) linked to a specific adolescent health project: Support to improve health outcomes by addressing the multi-sectoral determinants of health, with a particular focus on the economic, education, and demographic challenges in the northern regions of Cameroon, especially for adolescent girls (US$ 4 million IDA, US$3 million GFF Trust Fund).

Mozambique

- Investment case
  - Content: Detailed situational analysis and dedicated section on adolescent health, with strong emphasis on prevention of teenage pregnancy and prevention of early marriage. Adolescent and youth consulted in development of Investment Case.
  - Indicators: The M&E Matrix has several intermediate and outcome indicators relevant to adolescents, both in terms of health but also relevant to other sectors
  - Investment: No detail of allocation to adolescent health.
- Project Appraisal Document:
  - Content: Adolescents are mentioned as part of the goal to improve the utilization and quality of reproductive, maternal, child, and adolescent health and nutrition services (RMNACH-N), with an emphasis in the document on teenage pregnancy
  - Indicators: Adolescents are not mentioned in the indicators but included in the few RMNCAH indicators. Only one indicator that has a focus on adolescent health i.e. Percentage of secondary schools offering SRH services (information and contraceptive methods), based on visits by health professionals, at least monthly.
  - Investment: $15 million or 10% allocated to the indicator listed above

DRC

- Investment case
  - Content: Adolescent health is included but not comprehensively or analysed in depth
  - Indicators: Of eight indicators used to determine which provinces of DRC would be the focus of the investment case, one is focused on adolescent health: fertility rate among adolescents under 15 years of age.
  - Investment: Specific budget allocated to adolescent health projects: $2.5 million of GFF funding is allocated to improving the coverage and quality of reproductive health for adolescents and youth, just under 1% of a $2.6 billion budget. A total of $7.7 million is planned including resources from other development assistance partners.
- Project Appraisal Documents 2016
  - Content: No discussion of adolescent health, this PAD focuses on vital registration, essential medicines, procurement, and information management systems for the health and education sectors
  - Indicators: None
  - Investment: None
- Project Appraisal Document 2017:
  - Content: Holistic discussion of adolescent health
  - Indicators: One indicator, but not comprehensive: First time adolescent girls acceptance of modern contraceptives (number).
  - Investment: Allocation of GFF amount provided to additional family planning, nutrition, adolescent health, and in the RMNCAH PBF

package of services. The amount allocated to adolescent health is bundled into the 45% of the $390 million total proposed. It cannot be further disaggregated.

Ethiopia

- Investment case
  - Content: Adolescent health is included but not comprehensively
  - Indicators: Outcome indicator included for teenage pregnancy, but other reproductive health measures not disaggregated by age
  - Investment: No specific mention of investment (allocations/ disbursement) for any topic; Investment case is that national health plan
- Project Appraisal Document:
  - Content: Adolescent health mentioned including multi-sectoral issues such as FGM, GBV, community engagement
- Indicators: Disbursement linked indicator (Improve quality of adolescent health services) with expected achievements (milestones) which include publication of the Government Endorsed Adolescent and Youth Health Strategy (by 2017), Develop a Standard Package of Health Services for Schools (by 2018), Develop a training manual for healthcare providers to build their competencies in providing the minimum health services package to adolescents (by 2019), Increase in PHCs providing adolescent health services from baseline 60% to 75% (by 2020)
  - Investment: Allocation of GFF amount of US$5 million provided for linked indicator “Improve quality of adolescent health services”

Bangladesh

- Investment case – *not accessible*
- Project Appraisal Document 1 – health sector focus :
  - Content: Adolescent health is mentioned in situational analysis and then partially addressed via the Health, Nutrition and Population (HNP) programme to be delivered in schools
  - Indicators: No adolescent-specific health or outcome related indicators; but process indicators specific to implementing the HNP, which aims to improve adolescent health outcomes, with one process indicators specific to adolescent health and nutrition.
  - Investment: Allocation of GFF amount ($25 million USD) provided for school-based HNP programme for adolescents
- Project Appraisal Document 2 – secondary education focus:
  - Content: Adolescent health is mentioned in situational analysis and then partially addressed via the Adolescent Girls’ Programme (AGP) to be delivered in secondary schools
  - Indicators: No adolescent-specific health or outcome related indicators; but several process indicators specific to implementing the AGP
  - Investment: Allocation of GFF amount provided for AGP: $70 million USD, which is 14% of the total funding envelope.

Nigeria

- Investment case
  - Content: Adolescent are framed with the RMNCAH -N acronym, mentioned in contextual analysis, integrated in 6 strategic objectives with one adolescent specific one, although otherwise little detail provided
  - Indicators: No mention of adolescent specific indicator in Results Framework, but two indicators listed in in the body of the Report
  - Investment: Information does not provide disaggregated detail for adolescent health
- Project Appraisal Document:
  - Content: Adolescents are framed within the RMNCAH acronym. Context and humanitarian crisis in NE Nigeria a key part of the analysis and focus of services, which includes counselling for women, girls and boys who have experienced GBV
  - Indicators: Results Framework only notes one indicator that is directly relevant to adolescent girls e.g. Proportions of mothers aged 15 to 19 years of age who deliver in the last two years who receive skill birth attendance as well as one related to receiving counselling for GBV.
  - Investment: Insufficient information to assess what percentage if any, will be allocated to adolescent health, even as part of RMNCAH.

Guatemala

- Investment case
  - Content: Adolescent health mentioned only once in the situational analysis noting high prevalence of overweight and obesity
  - Indicators: None
  - Investment: none
- Project Appraisal Document:
  - Content: Addressing nutritional needs of adolescents is listed as part of the project objectives but this is then not mentioned again
  - Indicators: None
  - Investment: none
